# Management of Septic Open Abdomen in a Morbid Obese Patient with Enteroatmospheric Fistula by Using Standard Abdominal Negative Pressure Therapy in Conjunction with Intrarectal One

**DOI:** 10.1155/2015/293946

**Published:** 2015-12-08

**Authors:** Fahri Yetisir, A. Ebru Salman, Hasan Zafer Acar, Mehmet Özer, Muhittin Aygar, Gokhan Osmanoglu

**Affiliations:** ^1^Atatürk Research and Training Hospital, General Surgery Department, Ankara, Turkey; ^2^Atatürk Research and Training Hospital, Anesthesiology and Reanimation Department, Ankara, Turkey; ^3^General Surgery Department, Bozok University, Tokat, Turkey; ^4^Medical Park Private Hospital, General Surgery Department, Ankara, Turkey

## Abstract

*Introduction*. Management of open abdomen (OA) with enteroatmospheric fistula (EAF) in morbid obese patient with comorbid disease is challenging. We would like to report the management of septic OA in morbid obese patient with EAF which developed after strangulated recurrent giant incisional hernia repair. We would also like to emphasize, in this case, the conversion of EAF to ileostomy by the help of second Negative Pressure Therapy (NPT) on ostomy side, and the chance of new EAF occurrence was reduced with intrarectal NPT.* Case Presentation*. 62-year-old morbid obese woman became an OA patient with EAF after strangulated recurrent giant hernia. EAF was converted to ostomy with pezzer drain by the help of second NPT on ostomy. Colonic distention was reduced with the third NPT application via rectum. Abdominal reapproximation anchor (ABRA) system was used for delayed abdominal closure.* Conclusions*. Using the 2nd NPT on ostomy side may help in the maturation of the ostomy created in a difficult condition in an open abdomen. Using the 3rd NPT through rectum may decrease the chance of EAF formation by reducing the pressure difference between intraluminal pressure and extraluminal pressure in hollow viscera.

## 1. Introduction

Incisional hernias (IH) are a frequent complication after all abdominal surgery, with an incidence of 10–23% [1, 2]. Obesity and chronic diseases are predisposing factors for developing IH with the potential complication of small bowel obstruction and other morbidities [3]. IH enlarge over time and can cause serious complications like bowel obstruction due to incarceration or strangulation. In strangulated IH, bowel perforation and fistula formation may rarely develop preoperatively. Large and complicated hernias are a challenge for surgeons [4].

Open abdomen (OA) management is a life-saving and challenging strategy in situations such as the abdominal compartment syndrome (ACS), damage-control surgery, and severe generalized peritonitis [5, 6]. Enteric fistulas are one of the most devastating abdominal complications in abdominal surgery [7]. Management of patients with an open abdomen and an enteroatmospheric fistula (EAF) is very challenging. The mortality of EAF was as high as 70% in the past decades but is currently approximately 40% due to advanced modern intensive care and improved surgical techniques [8].

We would like to report management of OA in morbid obese patient with EAF which developed after strangulated recurrent giant incisional hernia repair. We would like to also emphasize in this case that converting EAF to ileostomy was achieved with pezzer drain by the help of synchronized second NPT with abdominal NPT on ostomy side, and the chance of new EAF occurrence during long OA period was reduced by decreasing colonic distention with intrarectal NPT, first time in the literature (Figure 4).

## 2. Presentation of Case

62-year-old morbid obese woman was admitted to the emergency department with complaint of abdominal pain, distention, constipation, and vomiting. For the last 4 days, the severities of complaints have increased. Her levels of consciousness and orientation were also worsened. In her past history, she had hypertension, diabetes mellitus (DM), hyperlipidemia, chronic lung disease (CLD), and depression. Up to now, she was operated on 8 times from the same side of the abdomen. First of all, she was operated on for acute cholecystitis 15 years ago; 1 year after this operation, subcostal IH developed. She has been operated on 6 times for IH. She was also operated on for ileus. During the last 2 years, she had irreducible giant ventral hernia and she went to emergency department 3 times for the same complaint.

In physical examination, Body Mass Index (BMI) was 47. On her abdominal examination, there were giant hernia and distention. Rebound and rigidity were positive at all quadrants of abdomen. She underwent emergent operation. There was severe adhesion and septic abdomen. During exploration necrosis and perforation of 70 cm ileum segment which was 50 cm proximal to ileocecal valve was seen. At first operation, septic abdomen was irrigated, necrotized ileum segment was resected, and end to end anastomosis was done. Due to giant hernia and severe peritonitis, delayed abdominal closure with Bogota bag was performed. She was transferred to ICU with vasopressor support.

**Figure 1:**
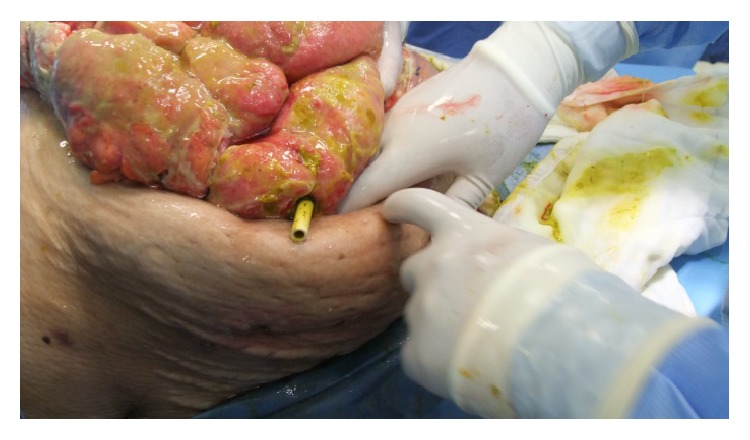
Pezzer drain is seen in EAF.

**Figure 2:**
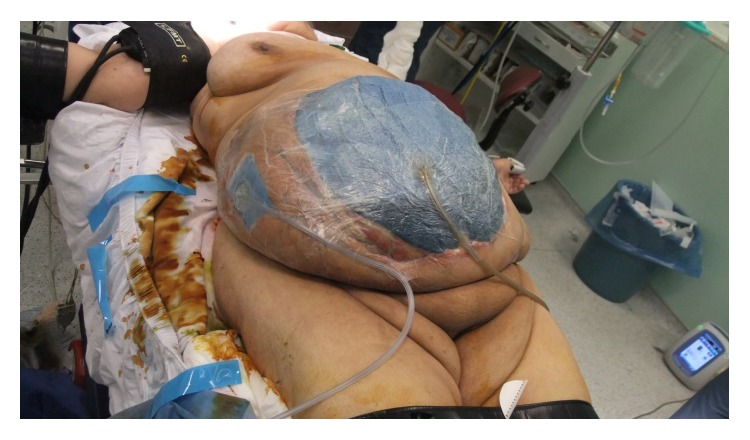
Two NPTs are seen.

At postoperative 9th day, after EAF development, she was consulted and transferred to our clinic. Her general condition, consciousness, and orientation were not well. She was mechanically ventilated. Her vital parameters, intra-abdominal pressure (IAP), SOFA score, and estimated mortality rate were shown in Table 1. She was in septic shock and mild metabolic acidosis (Table 2). She underwent emergent reoperation. There was very wide OA wound (70*∗*60 cm in diameter) with high output enteric fistula and severe visceral adhesion. All intestine was very edematous and fragile and according to new modified Björck classification OA score of the patient was 2c score [9]. All the intra-abdominal content was irrigated with saline. Perforation point of ileum was seen at the previous anastomosis side (Figure 1). A pezzer drain was inserted in the EAF and redirected to the place where ileostomy was planned to open. Glycerin-impregnated gauze was used around EAF and pezzer drain tube to support segmentation of ostomy side from OA wound. Two NPT systems were applied; one was standard abdominal NPT (Figure 2), and the second one was performed on the ileostomy opening where the EAF was directed with pezzer tube. Synchronized negative pressure was applied to both NPTs [10]. The second NPT on ostomy place was changed 3-4 times a day.

Ileostomy was maturated hardly step by step 4 days, after the first NPT application. Source control was achieved by converting EAF to loop ileostomy. All the vital and laboratory parameters of the patient were improved and intra-abdominal sepsis was resolved; vasopressor support was stopped. Distention of edematous bowel and the risk of new EAF formation were in progress. Synchronized intrarectal NPT with abdominal NPT [11] was introduced in order to decrease intracolonic distention and the difference between intracolonic pressure and intra-abdominal pressure.

**Figure 3:**
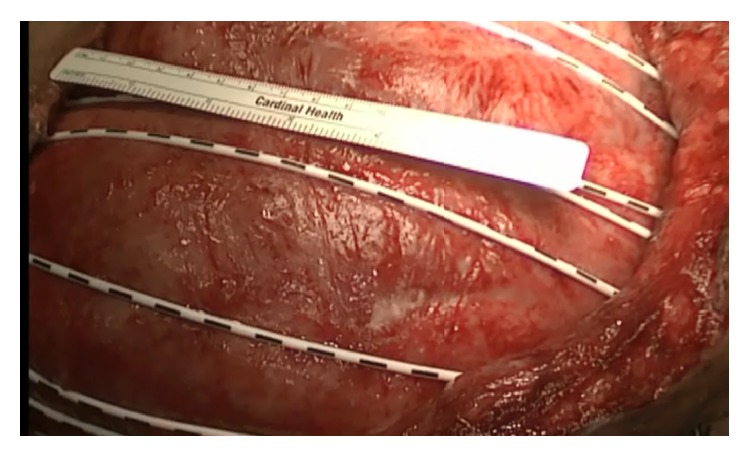
After ABRA application.

**Figure 4:**
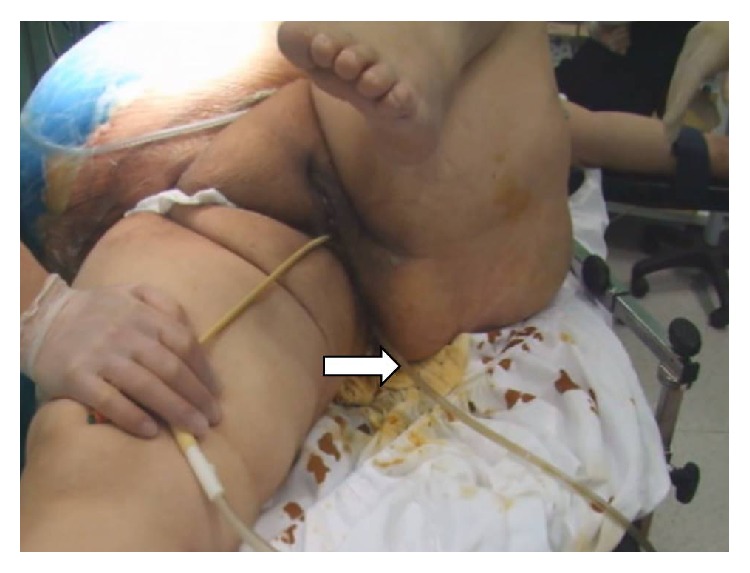
Intrarectal NPT was shown by arrow.

On postoperative 17th day, following intra-abdominal source control, abdominal reapproximation anchor (ABRA) system was added to facilitate the delayed OA closure (Figure 3). Abdominal drainage and ABRA arrangement were changed every 2–4 days. On the postoperative 75th day, delayed closure of OA was completed. She was discharged from the hospital on the postoperative 85th day with the suggestion of Lomotil 4*∗*1. Ileostomy had to be closed 4 months later due to prolapsed stoma. She started to defecate at postoperative 4th day and was discharged from the hospital at postoperative 6th day. There was no problem other than planned ventral hernia at 12-month follow-up period.

## 3. Discussion

There are lots of factors for recurrence of IH. Wound infection, obesity, DM, CLD, and closure technique are thought to be the most important risk factors [12]. In our case, she was elderly, morbid, obese patient with DM and CLD. She had complicated recurrent giant hernia, although she underwent operation for IH 6 times. Duration of strangulation is the most important determinant of the outcome regarding gut viability, resection-anastomosis rate, morbidity, and mortality [13]. Andrews noted a mortality rate of 1,4%, 10% and 21% when strangulated hernia presented within 24 hours, 24–48 hours, and after 48 hours, respectively [14]. In our case duration of strangulation was more than 96 hours and there was severe peritonitis with necrosis and perforation of small bowel at the first operation.

Severe peritonitis due to bowel necrosis with perforation is rare but debilitating complication of recurrent strangulated giant incisional hernia, like in our case. In septic condition, fascial closure of giant hernia (more than 10 cm) was not preferred. Skin approximation may be used to close if it is possible or if it is not possible, and OA management may be used with NPT. Management of open abdomen by using NPT was recommended in WSACS guideline [9].

Success rate and duration of OA management were influenced by obesity, OA wound size, SOFA score, Björck score of OA, and presence of EAF and comorbid diseases. According to our knowledge, wound size (70 cm *∗* 60 cm) of our case at first NPT application was the biggest one in the literature and she has more than one comorbid disease and SOFA score was 13 with 60% expected mortality accordingly.

There are multiple methods for controlling of EAF. For complicated open abdomen patient with EAF, a combination of different delayed abdominal closure methods is available [15]. For deeply localized EAF, fistula can be controlled by using Flexi-Seal in conjunction with NPT [16]. Diversion of the EAF after intubation with Malecot catheters, which is then tunneled through adjacent mobilized skin-subcutaneous flaps, can convert the EAF to a ECF, thereby simplifying wound care and potentially decreasing morbidity [17]. Our EAF controlling system was similar with this system in some respects. Our patient's EAF was converted to an ostomy in our case instead of ECF and NPT on the ostomy site was used during ostomy maturation. By using a second NPT on the ostomy site, we were able to eliminate the effect of abdominal NPT in order redirect enteric effluent toward the ostomy site instead of the intra-abdominal NPT site. At the end, loop jejunostomy was created because there is less complication during closure of loop stoma [18].

There are two fundamental hypotheses for development of EAF with the use of abdominal NPT; one is damage to bowel wall during frequent manipulation, and the second is as a result of the difference between intraluminal and extra luminal pressure. We used a nonadherent, silicon visceral cover to protect viscera as is standard for abdominal NPT. We also used a novel method to reduce the difference between intraluminal and extraluminal pressure and simultaneously decrease colonic distention by placing an intrarectal NPT device.

## 4. Conclusion

Using second synchronized NPT with abdominal NPT application on ostomy side may help to convert EAF to ostomy.

Intrarectal NPT application may reduce the chance of new EAF occurrence by decreasing the difference between intra- and extraluminal pressure in hollow viscera in a hostile open abdomen while using abdominal NPT.

## Figures and Tables

**Table 1:** Vital parameters and intra-abdominal pressure at admission to our clinic.

Fever	38°C
Hart rate	130/min
Blood pressure	80/55 mmHg
Intra-abdominal pressure	16 mmHg
SOFA score	13
Estimated mortality according to SOFA score	60%

**Table 2:** Laboratory value of patients before NPT application.

Biochemical analysis		Total blood count				Blood gas	
Glu.	183 mg/dL	WBC	25.000 K/uL	CRP	21 mg/dL (0–0.8)	pH	7.28
K	3.1 mmol/L	Hb	11.2 g/dL	INR	1.7	pCO2	41
Ca	7.1 mg/dL	Plt	233 K/uL	D-dimer	403 ng/mL (0–500)	PO2	78
LDH	287 U/L			Procalcitonin	23.5 ng/mL (<0.500)	HCO3	18
T. bilirubin	5.33 mg/dL					BE	−7.7
D. bilirubin	1.55 mg/dL						
Urea	104 mg/dL						
Creatinin	2.6 mg/dL						
Alb	1.6 g/dL						
